# Compact Eight-Element MIMO Antenna with Reduced Mutual Coupling and Beam-Scanning Performance

**DOI:** 10.3390/s22228933

**Published:** 2022-11-18

**Authors:** Ashfaq Ahmad, Dong-you Choi

**Affiliations:** Communication and Wave Propagation Laboratory, Department of Information and Communication Engineering, Chosun University, Gwangju 61452, Republic of Korea

**Keywords:** MIMO antenna, phased array antenna, SLL, diversity gain, envelope correlation coefficient

## Abstract

In this study, a multiple-input multiple-output (MIMO) antenna for wide scanning is designed. By adding slits to the patches, each element is intended to strengthen the isolation between the radiating elements. The proposed high isolation and wide scanning antenna combine to achieve the desired phased-array antenna. The array has a main beam pointing to the desired scanning region and a minimum side lobe level (SLL) in the undesired direction. A compact and linear eight-element array with an interelement spacing of λ/2 is designed and analyzed for beam scanning in the E-plane. Considering the worst case, the proposed array has a very low mutual coupling of (S21 = −24 dB), and it realizes a gain of 9.3 dBi and an SLL of 11 dB at a scanning angle of 70${}^{\circ }$. The antenna performance was studied in terms of the S-parameter, radiation patterns, beam-scanning performance, envelope correlation coefficient (ECC), diversity gain (DG), peak gain, and efficiency. A close agreement was observed between the simulated and measured results.

## 1. Introduction

Phased array antennas with beam-steering ability have identified numerous applications in communication technology [1,2]. Over the past decade, the interest in effective solutions for wide-beam scanning and high-gain design has increased. In [3,4], different approaches were used to achieve wide beam-scanning. However, a maximum scanning angle of 64${}^{\circ }$ was observed. In [5], phased-array antennas for 5G handsets were proposed to perform beam scanning in the elevation plan. A beam-switching technique based on a Rotman lens [6] or a Butler matrix [7] was proposed for mobile applications of millimeter waves. These techniques require a switch for beam switching. On a typical mobile phone chassis, only a single-layer array can be accommodated due to the bulky beam-switching technology. It is tricky to cover the whole spectrum as a result. The analog beamforming process uses a direct connection between chip and antenna ports, while the 29 GHz or lower band mm wave handsets are mostly focused on the antenna design.

A wide spectrum is available in the millimeter-wave (mm-wave) frequency range to meet the rising need for high data speeds in mobile communications [8]. However, high path loss is the main challenge in mm-wave communication (compared with sub-6 GHz cellular communication). Therefore, adaptive beam-forming/beam-scanning antennas are required to overcome these challenges and ensure connectivity. Similarly, MIMO technology is in high demand owing to its high transmission speed, resistance to multiple path fading, and large coverage area [9,10]. Similarly, mutual coupling is a non-negligible issue in MIMO antennas. For miniaturization, it is necessary to reduce the distance between the elements, resulting in improved mutual coupling and poor impedance and radiation performance. Mutual coupling can be reduced by spacing elements farther apart; however, this leads to an increase in the antenna size. Therefore, packing more elements with minimum spacing has attracted the attention of researchers. However, this is mainly discussed in terms of utilization [11], calculation [12,13], compensation [14,15], and the suppression of mutual coupling.

Different methods have been put forth to lessen mutual coupling. In [16,17,18], the surface waves were suppressed between the radiator using a defected ground structure (DGS); however, this caused a poor front-to-back ratio. Planner resonators such as the electromagnetic band-gap (EBG) [19,20,21], waveguide metamaterials [22], and parasitic resonate elements have been introduced to mitigate mutual coupling [23]; in [20], the mushroom-type EBG was interject between 2 × 1 patches, exhibiting good isolation of 10 dB but at the expense of high fabrication costs. Additionally, polarization-conversion isolators [24], neutralization lines [25,26,27], coupled resonator decoupling networks [28], slits in radiating elements [29], and interference cancellation chips [30] are some decoupling techniques. However, these techniques are limited to decoupling a two-element array. Other approaches include sticking stubs in the ground plane [31,32]. Similarly, an L- and T-shaped ground branch was added, leading to high isolation [33,34]. Slow-wave structures can be introduced between two elements to minimize mutual coupling by reducing the wavelength of the signal in the vicinity of the antenna and increasing the wavelength separation between them [35].

In the literature, the worst mutual couplings of the two-element array in [17,22] and [23] are −17, −12.6, and −16 dB, respectively, while the worst mutual coupling of the eight-element array that has been presented is −24 dB. Along with this significant improvement in mutual coupling and SLL of 11 dB, with a maximum achievable gain of 11.7 dBi at 0°, the suggested array provides enhanced beam-scanning performance from 0° to 70°.

This work proposed a compact, eight-element, switched-based, phased array MIMO antenna operating at 29 GHz for mobile phone application. Initially, an analysis was performed for the MIMO antenna consisting of straight elements, and each element of the array was bent at 45${}^{\circ }$ clockwise. To increase the current path, two diagonal slits were made on each antenna element of the proposed MIMO antenna. Unlike straight and bent element arrays, the proposed array exhibits maximum isolation, a minimum side-lobe level, and better beam-scanning performance. A summary of the simulated and measured results in terms of the S-parameters and scanning angles along with the desired realized gain was evaluated.

## 2. Design and Working Principle

The geometry and working principles of the proposed design are discussed in this section.

### 2.1. Geometry of Antenna

**Figure 1:**
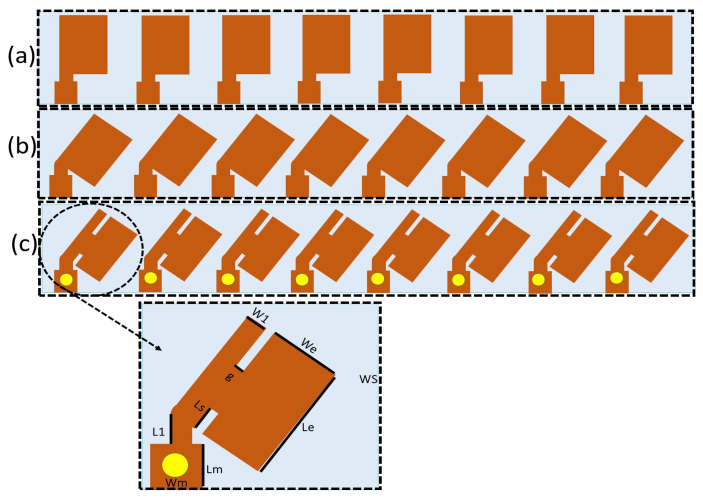
(**a**) Straight elements MIMO. (**b**) Bend elements MIMO. (**c**) Proposed eight-element MIMO.

**Figure 2:**
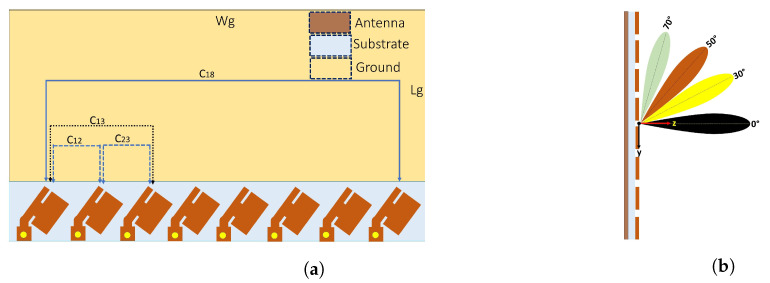
(**a**) Layout of proposed MIMO antenna (where C12 shows coupling between elements 1 and 2). (**b**) E-plane linear array with scanning schematic.

The 2-D layout of the designed eight-element MIMO antenna is depicted in Figure 1 and Figure 2. The CST microwave studio was used to perform simulation for this work The unit element is derived from a rectangular patch antenna element, the dimensions of which are determined using the transmission line theory [36]. The radiators are designed on a conductor that is backed by Roger RO4350B substrate, having an $\epsilon_{r}$ of 3.48, a loss tangent of 0.0037, and a thickness of 0.8 mm. Similarly, copper is used as a ground plane. The values of the antenna parameters are listed in Table 1. The lower part of the radiating element was connected to a coax probe. By varying the values of Lm and Wm, the unit cell is matched to 50 Ω line. For a specific resonance frequency, the effective resonant length (*Lre*) and width (*W*) are computed using the aforementioned theory [36].

(1) $$\begin{array}{c} L_{re}=\frac{c}{2f_{r}\sqrt{\frac{\epsilon_{r}+1}{2}+\frac{\epsilon_{r}-1}{2}{\left(1+12\frac{h}{w}\right)}^{-0.5}}}-2\Delta L \end{array}$$

(2) $$\begin{array}{c} W=\frac{1}{2f_{r}\sqrt{\mu_{o}\epsilon_{o}}}\sqrt{\frac{2}{\epsilon_{r}+1}} \end{array}$$

Furthermore, *h* represents the substrate thickness, *c* represents the speed of light, $f_{r}$ represents the resonance frequency, and ΔL represents the differential change in the length caused by fringing. Moreover, the free-space permittivity, relative permittivity, and permeability of free space are $\epsilon_{o}$, $\epsilon_{r}$, and $\mu_{o}$, respectively.

The optimized architecture of the proposed design consists of an eight-element beam-switch-based MIMO antenna as an example to study the mutual coupling effect, as shown in Figure 1, while Figure 2b shows the E-plane scanning schematic of the proposed linear MIMO antenna. The MIMO antenna is designed to operate at a frequency of 29 GHz. The overall size was 43.7 × 118 mm${}^{2}$. Each of the eight antenna elements are optimized to radiate at 29 GHz and are excited by a separate 50 Ω coax connector. To study the beam-scanning performance and mutual coupling effect, we used an eight-element linear array with an inter-element spacing of approximately $\lambda_{o}$/2, where $\lambda_{o}$ is the free-space wavelength at 29 GHz. Figure 2a presents the proposed design and a mobile chase.

**Figure 3:**
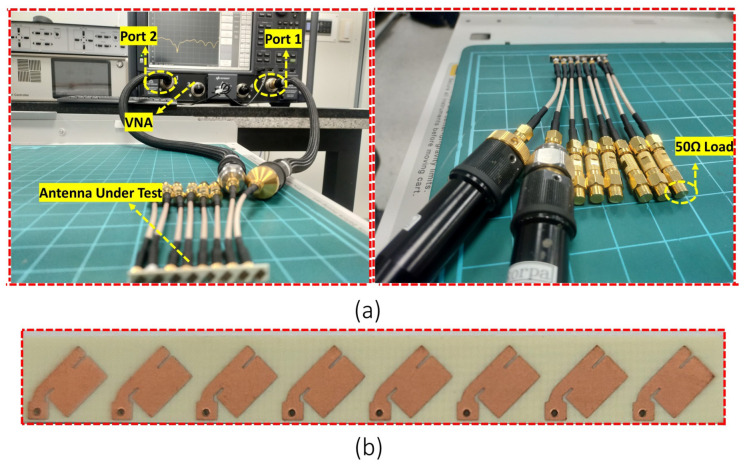
(**a**) Measurement setup for S-parameter. (**b**) Fabricated design.

The eight-element MIMO antenna that has been proposed is manufactured and measured. Taking into consideration the worst case scenario, only closely spaced elements were measured and evaluated. A PNA network analyzer with model number N5227B (frequency range of 10 MHz to 67 GHz) was used to measure the S-parameters. To measure S11 and S12, elements 1 and 2 were connected to the network analyzer, whereas the remaining six elements were terminated with a 50 Ω load. The measurement setup for the s-parameter and prototype of the proposed design are shown in Figure 3.

### 2.2. Working Principle

The main challenge in this work is to design an array with beam-steering ability, high realization gain in the desired frequency band, a low side-lobe level, and reduced mutual coupling. To overcome these challenges, an eight-element linear array is designed. In [37], 40 dB of isolation is observed by introducing the slot in the ground plane. However, this approach had a deleterious effect on the required radiated power since it caused a severe back radiation pattern by vanishing half of the power into free space. By adding two slots to the patches and rotating them 45${}^{\circ }$ clockwise, it was possible to notice high isolation between nearby elements in the proposed design. The slots and stub (left part of the radiator) act as a capacitor and inductor, respectively, and as a parallel LC equivalent circuit. The slots increase the current path, leading to high isolation at the desired frequency of 29 GHz.

## 3. Mutual Coupling Effects

As explained earlier, mutual coupling always exists between the closely placed antenna elements. Figure 2a presents a linear array with slits in the patches, in which $C_{ij}$ shows the mutual coupling between the *i*th and *j*th elements. The coupling between two adjacent elements often dominates, that is, $C_{12}$ is generally much larger than $C_{13}$, and thus $C_{18}$ is the smallest and negligible. Thus, for the analysis, only $C_{12}$, $C_{23}$, $C_{34}$, and $C_{45}$ were considered in the proposed array design.

To examine the mutual coupling effect, we chose a switched-based phased array as an example. Specifically, a rectangular patch antenna was used as the radiating element. Figure 2 shows the setup model that was used to depict mutual coupling, where eight identical radiating elements are accommodate at a distance of 0.5$\lambda_{o}$ and $\lambda_{o}$ is the free-space wavelength; each element is excited with a 50 Ω coax feedline. The dimensions of each radiating patch were the same and are presented in Table 1.

### 3.1. Current Distribution

**Figure 4:**
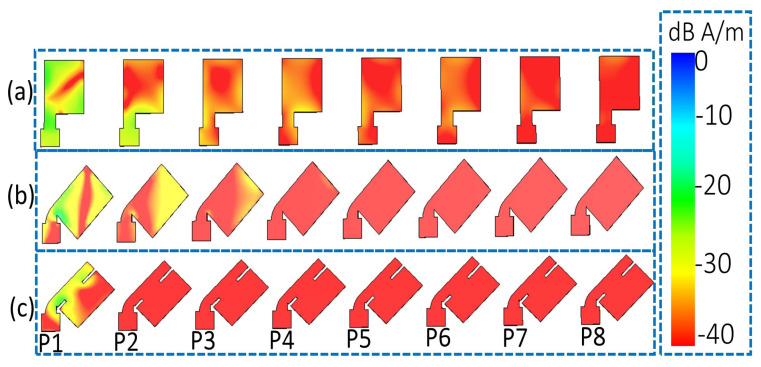
Surface current distribution of (**a**) straight elements, (**b**) bend elements, and (**c**) proposed eight-element MIMO antenna.

To better understand the mechanism of the mutual coupling reduction, the surface current of the designed array is depicted in Figure 4. The first element (P1) was excited, whereas the remaining elements were terminated with a 50Ω load. Initially, element 1 has more influence on element 2 and gradually decreases for elements 3 and 4, as shown in Figure 4a. However, as seen in Figure 4b, when the radiating elements are rotated 45° clockwise, element 1 dominates element 2 and has no impact on element 3. Finally, by introducing two diagonal slits in the radiating element, which increases the length of the surface current, it can be perceived from Figure 4c that element 1 has a negligible effect on element 2, which shows better isolation between the closely packed radiating elements.

### 3.2. S-Parameter

**Figure 5:**
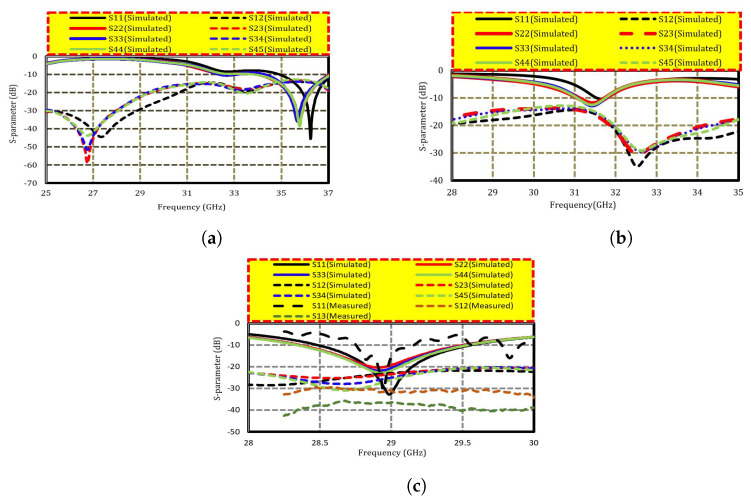
S-parameter of (**a**) straight elements, (**b**) bend elements, and (**c**) proposed eight-element MIMO antenna.

The simulated s-parameters of the straight-element array are depicted in Figure 5a. Because the array is symmetric, only the transmission and reflection coefficients of port 1–4 are shown here (which correspond to the four elements on the left side of the array), see Figure 1c). The array resonates at 36.1 GHz with a transmission coefficient of approximately −14 dB. However, to further enhance the isolation, each segment of the MIMO array is rotated 45° clockwise, as depicted in Figure 1b. The scattering parameter of the bend antenna array is presented in Figure 5b, where the resonance frequency is shifted to 31.8 GHz, and S11 is close to −11 dB, which shows the mismatch of the antenna. In this case, a mutual coupling of −15.5 dB is observed. However, mutual coupling is further diminished by optimizing the orientation of the radiating patches and introducing slots in the radiators. The overall dimensions of the rectangular patch were 3.62 × 2.7 mm${}^{2}$. Each element was excited with a 50 Ω coax fedline. The proposed design shows S11 close to −33 dB, which guarantees perfect matching, and it has a mutual coupling of more than 24 dB. The dimensions of the lower notch are 1.22 × 0.18 mm${}^{2}$, whereas the dimensions of the upper notch are 0.7 × 0.18 mm${}^{2}$, which further reduces the coupling to −35 dB. The S-parameter of the intended design is measured and equated with the simulated results; for convenience, only the measured values of S11, S12, and S13 are presented in Figure 5c. For S11, a measured reflection coefficient of −30 dB is observed; similarly, S12 of −31 dB is observed, showing high isolation between the closely spaced elements. When measuring S11 and S12, the remaining six elements were terminated with a 50 Ω fedline.

### 3.3. Envelope Correlation Coefficient and Diversity Gain

**Figure 6:**
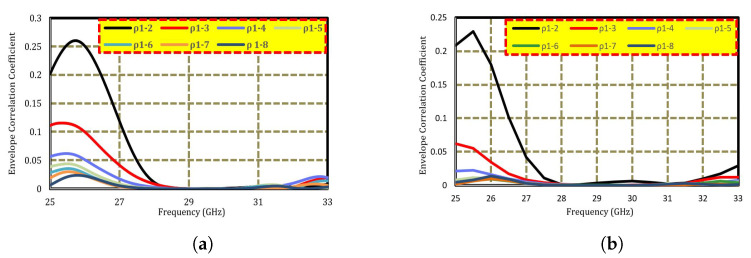
Envelope correlation coefficient of the proposed MIMO antenna using (**a**) S-parameters and (**b**) radiation patterns.

To further demonstrate the performance of the proposed antenna, the simulated envelope correlation coefficient (ECC) of the MIMO antenna is shown in Figure 6a,b. The ECC was evaluated between ports i and j, denoted by $\rho_{ij}$. To validate the effectiveness of the design and determine the quality of the uncorrelated channel, it must be calculated for the incoming signals. MIMO systems with high ECC values result in minimal isolation and high correlation, which degrade antenna performance. Different approaches can be used to determine the ECC, such as using the received signal envelope, an S-parameter, or far-field radiation.

(3) $$\begin{array}{c} \rho_{ij}=\frac{|S_{ii}*S_{ij}+S_{ji}{*Sjj|}^{2}}{\left(1-\right|S_{ii}{|}^{2}-{\left|Sji\right|}^{2})(1-|S_{jj}{|}^{2}-{\left|Sji\right|}^{2})} \end{array}$$

The ECC of the proposed MIMO antenna, calculated using the simulated S-parameters, is shown in Figure 6a. To further validate the results, the ECC was evaluated using the radiation pattern, as shown in Figure 6b.

(4) $$\begin{array}{c} \rho_{ij}=\frac{|\int \int_{0}^{4\pi }\left[F_{i}\left(\theta ,\phi \right)\times F_{j}\left(\theta ,\phi \right)d\Omega \right]{|}^{2}}{[\int \int_{0}^{4\pi }{\left[F_{i}\left(\theta ,\phi \right)\right|}^{2}d\Omega |[\int \int_{0}^{4\pi }{\left[F_{j}\left(\theta ,\phi \right)\right|}^{2}} \end{array}$$

where Fi and Fj represent the far-field radiation characteristics of ports i and j, respectively. In the proposed MIMO antenna, the simulated ECC was below 0.3. This shows that the antenna components interact minimally, making them a feasible candidate for 5G mobile communication.

**Figure 7:**
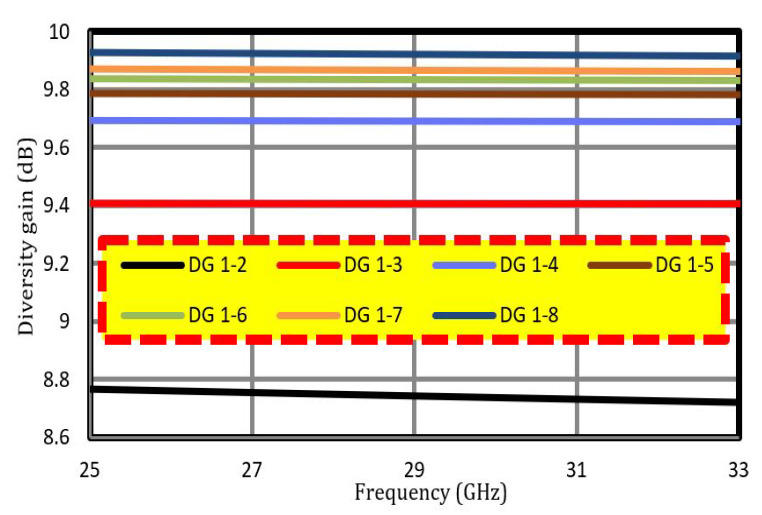
Diversity gain of the proposed MIMO antenna.

The diversity gain (DG) of MIMO antennas is an important parameter to consider. This is indicative of the reliability of the MIMO systems. Figure 7 presents the diversity gain (DG) between Ant 1-2, Ant 1-3, and so on. The value of DG for Ant1-2 to Ant1-8 was >8.7 dB for the desired band. The following equation can be used to calculate the DG:

(5) $$\begin{array}{c} DG=\sqrt{1-{\left(ECC\right)}^{2}}\; \end{array}$$

### 3.4. Efficiency and Peak Gain

**Figure 8:**
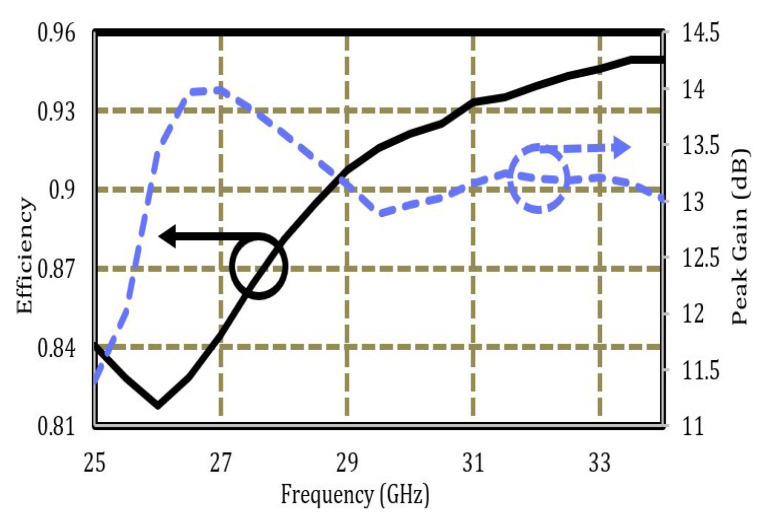
Efficiency and peak gain.

The simulated efficiency and peak gain are shown in Figure 8. An efficiency close to 90% was observed in the desired frequency band, which is adequate for 5G communication. Similarly, a peak gain of 13.96 dBi is observed at 26.5 GHz, and a small variation of gain lies within 12–13.96 dBi. A flat gain of more than 13 dBi was achieved within the operating bandwidth, which makes the proposed design a better candidate for mmWave communication.

### 3.5. Radiation Patterns

**Figure 9:**
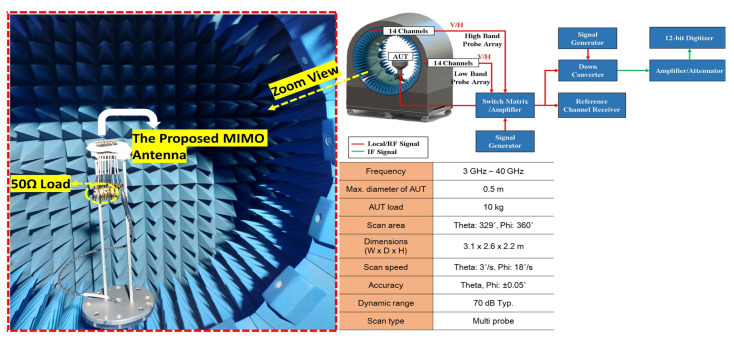
Radiation pattern measurement setup.

**Figure 10:**
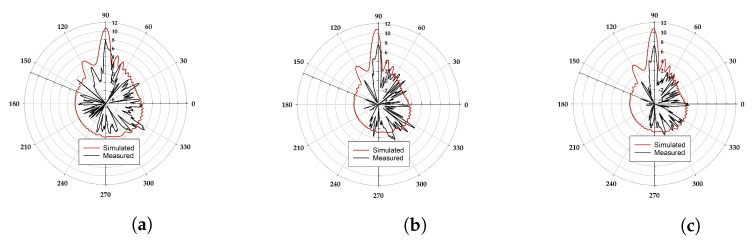
Simulated and measured radiation pattern. (**a**) 28.5 GHz, (**b**) 29 GHz, and (**c**) 29.5 GHz.

The radiation pattern measurement setup and detailed parameters are shown in Figure 9. The simulated and measured radiation patterns of the proposed eight-element MIMO antenna are shown in Figure 10. The antenna radiation performance was measured at 28.5, 29, and 29.5 GHz. However, only one measured port (P1) is provided due to design simplicity and symmetry concerns. Port 1 is connected to the source, while the other seven ports (P2-8) are terminated with a 50 Ω load. Figure 10 shows the measured peak gain of the proposed MIMO antenna. The disagreement between the measured and simulated results may be due to the inaccuracy of the substrate permittivity and the connectors.

## 4. Beamscanning Performance

**Figure 11:**
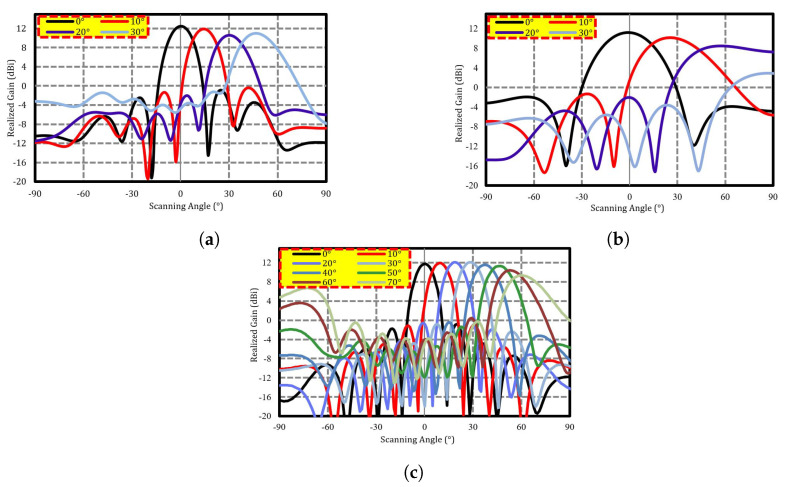
Beamforming in (**a**) straight elements MIMO, (**b**) bend elements MIMO, and (**c**) proposed elements (refer to Figure 1).

As illustrated in Figure 1, the proposed MIMO antenna was designed and fabricated using Roger 4350 B as a substrate with a full-ground plane. The E-plane, a linear array with a scanning schematic, is shown in Figure 2b. Initially, the straight element array, i.e., from Figure 1, operating at 36 GHz, had good beam-scanning performance and a gain level of 11.8 dBi from 0–40° to as shown in Figure 11a. To achieve better beam steering with acceptable gain and low side lobe levels, straight elements were bent to 45${}^{\circ }$, and diagonal slots were introduced in the radiators. Equation (6) is used to find the phase shift π for the desired pointing angle θ and is presented below.

(6) $$\begin{array}{c} \psi =2\pi (\frac{d}{\lambda })\sin\theta \end{array}$$

where d, λ, and θ are the element spacing, wavelength, and pointing angle, respectively. Table 2 lists the pointing angles and the input phase shift.

Figure 11 shows the simulated realized gain for different scanning angles. It is observed that the proposed design exhibits better beam-steering behavior with acceptable gain levels at different scanning angles. It is observed that the realized gain levels are almost constant from 0° to 70°, with a small variation of less than 2.5 dBi. The worst realized gain of 9.3 dBi is obtained at a scanning angle of 70°, making this design acceptable for 5G mobile handset communication, compared to the straight element array where the worst gain of 5.7 dBi is obtained at 60°. It can be observed that 11.7 dBi antenna gains are obtained in the main beam direction. It should be noted that the beam-steering characteristics of the antenna for both the positive and negative scanning angles are symmetrical. In the measurement of the proposed design, each element was connected to a 50 Ω connector, which was further connected to a phase shifter.

**Figure 12:**
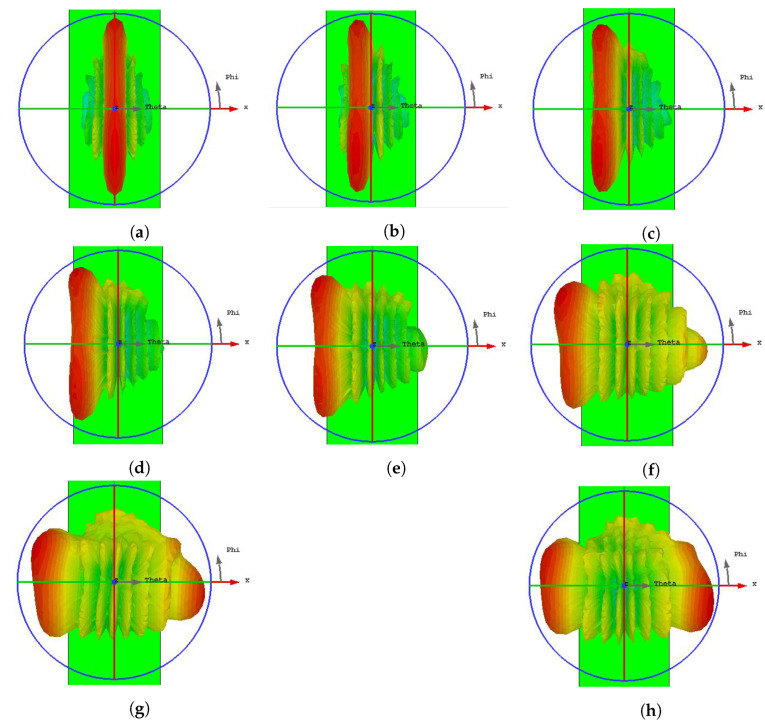
3D beam scanning of the proposed MIMO array at (**a**) 0°, (**b**) 10°, (**c**) 20°, (**d**) 30°, (**e**) 40°, (**f**) 50°, (**g**) 60°, and (**h**) 70°.

Figure 12 shows the simulated 3D radiation patterns of the proposed beam-steerable array in different states at 29 GHz. The radiation patterns at the other frequencies of 28–30 GHz were similar to those in Figure 12. With different input phases, the scan range varied from 0° to 70°. The wide scan range was caused by the beam width of the diagonal slit-shaped radiator element. The far field of the proposed array is constructed using the far field of a radiating element with slits. Therefore, array beam scanning is related to the radiating elements instead of the long chassis edge.

A comparison of the proposed eight-element MIMO antenna with other methods reported in the literature is summarized in Table 3. In [16], a defected ground structure was used for mutual coupling reduction, which caused a reduction in coupling but led to an improvement in the backward radiation. In [17], an improvement of 10 dB isolation was achieved using split-ring resonators.

In [22] a slit-based waveguide was used to improve the isolation between two H-plan-coupled patches, and an improvement of approximately 6 dB was observed in the desired frequency band. A slotted mender line was used to minimize the mutual coupling between the radiators in [23]. In all of the above cases, mutual coupling is observed between only two elements, without considering the beam-scanning performance. However, in [6], a substrate-integrated waveguide was used to reduce the mutual coupling between four elements, and the proposed design achieved a gain of 10.3 dB with a maximum beam scanning range of 76°. Similarly, in [38] different configurations were used to analyze the mutual coupling effect on the antenna performance, and it was concluded that isolation increases active reflection, which further leads to the maximization of the scanning angle. It was observed that adding notches to the back of the array improved the isolation by 15 dB. A multilevel dipole with beam-steering ability was proposed in [39], which has a scanning range of 42°, realizes a gain of 9 dBi, and operates in a low-frequency band of 3.5 GHz. In the proposed design, an eight-element array was designed at a center frequency of 29 GHz. Isolation is improved by introducing slots into the patches. A maximum scanning angle of 70° was observed for the proposed phased-array antenna.

## 5. Conclusions

In this paper, the design of a 5G phased-array antenna was presented. Initially, an antenna array of straight elements was evaluated; each element was rotated in a 45° clockwise direction, and finally, diagonal slots were made in each radiating element. It was observed that the high mutual coupling in the first two cases caused a high reduction in the beamforming gain and limited the maximum scanning angle of the phased array antenna. However, in the case of the proposed design, the mutual coupling improved to −24 dB and better scanning performance was observed up to 70° with a maximum realize gain of 11.7 dBi at 0°.

## Figures and Tables

**Table 1:** Parameters of proposed unit cell and MIMO antenna (all dimensions are in mm).

Parameters	Dimension (mm)	Parameters	Dimensions (mm)
wg	43.7	Wm	1.3
lg	118	Lm	1.2
Ws	5.3	L1	1.2
Le	3.62	W1	0.7
We	1.82	g	0.18
Ls	0.7		

**Table 2:** Pointing angle vs. phase shift.

Pointing Angle (θ)	Phase Shift (ψ)	Pointing Angle (θ)	Phase Shift (ψ)
0	0	40	115.70
10	31.25	50	137.88
20	61.56	60	155.88
30	90	70	169.14

**Table 3:** Comparison table.

Reference	Technique Used	Number of Elements	Center Frequency (GHz)	Realize Gain	Spacing between Elements	Isolation Improved	Maximum Scanning Angle
[16]	DGS	2	9.2	NA	0.25$\lambda_{o}$	16	NA
[17]	SRR	2	5	NA	0.25$\lambda_{o}$	10	NA
[22]	Waveguide Metamaterial	2	3.5	NA	0.125$\lambda_{o}$	6	NA
[23]	Slotted Meander-Line	2	4.8	NA	0.38$\lambda_{o}$	16	NA
[6]	SIW	4	33	10.3	NA	NA	76°
[38]	Notches in Ground Plane	8	28	14	0.5$\lambda_{o}$	15	60°
[39]	Multilevel Antenna	4	3.5	9	0.32λ	15	42°
Proposed Design	Slots in the Patches	8	29	11.7	0.5$\lambda_{o}$	14.7	70°

## Data Availability

Not applicable.
